# Upregulation of FOXM1 leads to diminished drug sensitivity in myeloma

**DOI:** 10.1186/s12885-018-5015-0

**Published:** 2018-11-21

**Authors:** Chunyan Gu, Xuefang Jing, Carol Holman, Ramakrishna Sompallae, Fenghuang Zhan, Guido Tricot, Ye Yang, Siegfried Janz

**Affiliations:** 1 The Third Affiliated Hospital, Nanjing University of Chinese Medicine, Nanjing, 210023 China; 2 Key Laboratory of Acupuncture and Medicine Research, Ministry of Education, Nanjing University of Chinese Medicine, Nanjing, 210023 China; 3 Department of Pathology, The University of Iowa Roy J. and Lucille A. Carver College of Medicine, Iowa City, Iowa 52242 USA; 4 Iowa Institute for Genetics, The University of Iowa Roy J. and Lucille A. Carver College of Medicine, Iowa City, Iowa 52242 USA; 5 Department of Internal Medicine, The University of Iowa Roy J. and Lucille A. Carver College of Medicine, Iowa City, Iowa 52242 USA; 6 Holden Comprehensive Cancer Center, The University of Iowa Roy J. and Lucille A. Carver College of Medicine, Iowa City, Iowa 52242 USA; 7 Department of Medicine, Medical College of Wisconsin, Milwaukee, WI 53213 USA

**Keywords:** Plasma-cell neoplasm, Targeted cancer therapy, Small-drug inhibitor, Cellular senescence

## Abstract

**Background:**

Following up on previous work demonstrating the involvement of the transcription factor forkhead box M1 (FOXM1) in the biology and outcome of a high-risk subset of newly diagnosed multiple myeloma (nMM), this study evaluated whether *FOXM1* gene expression may be further upregulated upon tumor recurrence in patients with relapsed multiple myeloma (rMM). Also assessed was the hypothesis that increased levels of FOXM1 diminish the sensitivity of myeloma cells to commonly used myeloma drugs, such as the proteasome inhibitor bortezomib (Bz) and the DNA intercalator doxorubicin (Dox).

**Methods:**

*FOXM1* message was evaluated in 88 paired myeloma samples from patients with nMM and rMM, using gene expression microarrays as measurement tool. Sources of differential gene expression were identified and outlier analyses were performed using statistical methods. Two independent human myeloma cell lines (HMCLs) containing normal levels of FOXM1 (FOXM1^N^) or elevated levels of lentivirus-encoded FOXM1 (FOXM1^Hi^) were employed to determine FOXM1-dependent changes in cell proliferation, survival, efflux-pump activity, and drug sensitivity. Levels of retinoblastoma (Rb) protein were determined with the assistance of Western blotting.

**Results:**

Upregulation of *FOXM1* occurred in 61 of 88 (69%) patients with rMM, including 4 patients that exhibited > 20-fold elevated expression peaks. Increased FOXM1 levels in FOXM1^Hi^ myeloma cells caused partial resistance to Bz (1.9–5.6 fold) and Dox (1.5–2.9 fold) in vitro, using FOXM1^N^ myeloma as control. Reduced sensitivity of FOXM1^Hi^ cells to Bz was confirmed in vivo using myeloma-in-mouse xenografts. FOXM1-dependent regulation of total and phosphorylated Rb agreed with a working model of myeloma suggesting that FOXM1 governs both chromosomal instability (CIN) and E2F-dependent proliferation, using a mechanism that involves interaction with NIMA related kinase 2 (NEK2) and cyclin dependent kinase 6 (CDK6), respectively.

**Conclusions:**

These findings enhanced our understanding of the emerging FOXM1 genetic network in myeloma and provided preclinical support for the therapeutic targeting of the FOXM1-NEK2 and CDK4/6-Rb-E2F pathways using small-drug CDK and NEK2 inhibitors. Clinical research is warranted to assess whether this approach may overcome drug resistance in FOXM1^Hi^ myeloma and, thereby, improve the outcome of patients in which the transcription factor is expressed at high levels.

**Electronic supplementary material:**

The online version of this article (10.1186/s12885-018-5015-0) contains supplementary material, which is available to authorized users.

## Background

With an estimated 30 thousand cases annually, newly diagnosed multiple myeloma (nMM) is the second most common blood cancer in the United States [1]. MM is a neoplasm of immunoglobulin-producing plasma cells that reside in the bone marrow. Quintessential disease manifestations include serum M-spikes (paraproteins), lytic bone lesions, hypercalcemia and renal insufficiency [2]. Owing to both newly developed myeloma drugs and the continuous refinement of therapeutic regimens that combine high-dose chemotherapy (HDT) with autologous hematopoietic stem cell transplantation (ASCT), the outcome for patients with nMM has significantly improved in recent years [3] – making it possible, at long last, to cure a tangible number of patients [4]. However, in the great majority of cases, following a period of successful therapy, myeloma relapses as a drug-refractory aggressive disease that leaves few, if any, therapeutic options. The unmet medical need of relapsed multiple myeloma (rMM) warrants dedicated research to enhance our understanding of the underlying pathways and identify new molecular targets for the design and testing of novel treatment approaches.

Although potent myeloma treatments, particularly proteasome inhibitors (PIs) and immunomodulatory drugs (IMiDs), have given the means to durable responses and prolonged survival of patients with myeloma, the inevitable relapse with drug-resistant disease is all too common. The root cause of acquired drug resistance in rMM is poorly elucidated, yet increasing evidence points to the involvement of a complex population dynamic of neoplastic myeloma growth [5] characterized by competition of co-existing tumor cell clones that eventually give rise to a dominant treatment-refractory clone able to thrive under conditions of strong drug-induced selective pressure. Genetic and genomic studies have shown that the evolutionary process sketched out above is driven by point mutations in drug response and other genes [6], copy number alterations that can abrogate tumor suppressor pathways [7] and changes in the epigenome that can reshape phenotypic and functional features of myeloma cells by virtue of affecting gene expression [8]. Another driver of the intricate pathophysiology of rMM is the bone marrow microenvironment, which provides tumor-promoting interactions with resident bone cells and the innate and adaptive immune system [9]. Increased cancer stemness may also be involved [10], the annoying elusiveness of bona fide myeloma stem cells notwithstanding.

Heartened by recent findings on the key role of the transcription factor, forkhead box M1 (FOXM1), in the genetic network of myeloma [11], we here continue with previous studies on the impact of FOXM1 in nMM [12] and rMM [13] and show that acquisition of drug resistance may be an important mechanism by which FOXM1 facilitates disease progression and relapse. Upregulation of FOXM1 rendered human myeloma cell lines (HMCLs) in continuous in vitro culture partially resistant to the PI, bortezomib (Bz), and the DNA intercalator, doxorubicin (Dox). In agreement with that was the in vivo result that enforced expression of FOXM1 in a HMCL designated CAG reduced the sensitivity of myeloma-in-mouse xenografts to Bz. We also provide evidence that FOXM1, presumably by virtue of its interaction with the retinoblastoma (Rb) cell cycle progression and tumor suppressor protein, promotes β-galactosidase (β-gal^+^) activity in myeloma – the classic Rb-regulated phenotype of cellular senescence that is mechanistically linked to relapsed cancer by means of acquired drug resistance, cancer dormancy and cancer stemness [14]. The result of this study adds strength to the contention that the therapeutic targeting of FOXM1 may benefit patients with myeloma in which the transcription factor is strongly expressed.

## Methods

### *FOXM1* expression in myeloma and treatment of patients with myeloma

Levels of *FOXM1* mRNA in myeloma cells were determined using Affymetrix U133Plus 2.0 microarrays (Santa Clara, CA) as previously described [15, 16]. Statistical analysis of microarray data relied on GCOS1.1 software (Affymetrix, Santa Clara, CA). Patients at UAMS were treated using the Total Therapy 2 regimen, the backbone of which is high-dose melphalan therapy (HDT) and autologous stem cell transplantation (ASCT). Half of the patients received thalidomide both during intensive therapy and as maintenance therapy. The therapeutic approach to relapsing disease was not uniform and depended mainly on the time to relapse, the pace of relapse (slow versus aggressive), the presence or absence of organ dysfunction, and the patient’s overall health status, physical and mental fitness and treatment preference.

### Human myeloma cell lines (HMCLs), myeloma drugs, and other agents

Four IgA-producing HMCLs, designated CAG, XG1, H929 and ARP1, were included in this study. The identity of the cell lines was validated as previously described [12], using chromosomal translocation status and gene expression spikes as main parameters. Cells were propagated in vitro at 37 °C and 5% CO_2_ using RPMI1640 cell culture medium (Gibco) supplemented with 10% heat-inactivated fetal bovine serum (Atlanta Biologicals) and antibiotics (100 units/mL penicillin and 100 μg/mL streptomycin, Sigma). In some experiments, CAG and XG1 cells over-expressing FOXM1 (FOXM1^Hi^) were compared to cells containing normal amounts of FOXM1 (FOXM1^N^) [12]. In other experiments, H929 and ARP1 cells in which FOXM1 expression had been knocked down using shRNA (FOXM1^Lo^) were compared to parental FOXM1^N^ cells [12]. Chemicals including myeloma drugs were purchased from Sigma (doxorubicin [Dox], thiostreptone [TS]), Millennium Pharmaceuticals (bortezomib [Bz]), or Invitrogen (propidium iodide, RNase A).

### In vitro assays using HMCLs

For cell cycle analysis, cells were fixed in ice-cold ethanol (1 h, 4 °C), washed in PBS, re-suspended in propidium iodide (PI) solution (40 μg/ml, 3 h, 4 °C) supplemented with 50 μl RNase A (10 μg/ml), and evaluated by flow cytometry using a FACScan (Becton Dickinson, San Jose, CA). For determination of clonogenicity, 10^4^ myeloma cells were seeded in soft-agar plates (0.5 ml RPMI1640 supplemented with 0.33% agar and 10% FBS) and grown for 2 weeks at 37 °C and 5% CO_2_ – in some cases exposed, during week 2, to myeloma drugs. Myeloma clones, defined as tight aggregates of ≥40 tumor cells, were enumerated on digital images of soft-agar plates analyzed with the help of Image J. For measurement of proliferation and viability, cells were counted using a hemocytometer and evaluated for exclusion of trypan blue (0.4% dye in PBS, pH 7.3), respectively. For determination of apoptosis, the flow-cytometric Annexin V APC assay (eBioscience, San Diego, CA) was used according to the manufacturer’s instructions. For determination of drug-efflux capacity, the flow-cytometric eFluxx-ID™ multidrug resistance assay was employed. MCF7 human breast cancer cells were included as benchmark. For determination of senescence-associated β-galactosidase (β-gal) activity, a kit from Cell Signaling Technology (Cat# 9860) was used. Briefly, cells were fixed (15 min), washed (PBS) and incubated in β-gal staining solution overnight at 37 °C.

### Western blotting

Protein levels were determined by Western analysis using antibodies to FOXM1, Rb and pRb obtained from Santa Cruz Biotechnology (FOXM1, sc-500) or Cell Signaling Technology (Rb, 9309; pRb, 8515). Cells were lysed with assistance of the Mammalian Cell Extraction kit (K269–500) from Biovision, Milpitas, CA. 10-μg samples of protein were fractionated on 4–12% SDS-PAGE gels, followed by transfer to nitrocellulose membranes blocked with 5% non-fat dry milk in Tris-buffered saline containing 0.05% Tween-20. Incubation with primary antibodies occurred overnight at 4 °C. Proteins were visualized using HRP-conjugated secondary antibody and SuperSignal West Pico (Pierce, Rockford, IL). Membranes were subsequently stripped and re-probed for β-actin (Santa Cruz Biotechnology, sc-47778), which served as loading control.

### HMCL xenografting in NSG mice

To compare the drug response in CAG myeloma cells expressing normal and elevated FOXM1 levels, respectively, 2 × 10^6^ FOXM1^N^ and FOXM1^Hi^ cells were injected subcutaneously (SC) into the right or left flank of NSG mice (Jackson Laboratory, Bar Harbor, Maine). Viability of FOXM1^N^ and FOXM1^Hi^ cells was comparable (≥90%). Seven days later, one group of mice was treated with bortezomib (1 mg/kg) administered intraperitoneally (IP) twice weekly. Another group of mice, designated untreated control, was injected with drug vehicle, normal saline (0.9% sodium chloride). In all cases, tumor growth was measured using a pair of calipers. Mice were sacrificed for humane reasons using CO_2_ asphyxiation when tumors reached 20 mm in diameter. All studies were approved under protocol 1301010 of the Institutional Animal Care and Use Committee of the University of Iowa.

### Statistical analysis

Two-tailed Student’s t-test was used to compare two experimental groups, employing parametric or non-parametric methods in case data were normally distributed or not, respectively. One-way analysis of variance (ANOVA) was used to evaluate more than two groups. To compare drug responses in mice, linear regression and AUC (area under curve) determination were used. For all analyses, the GraphPad Prism 7 software package (La Jolla, CA) was employed and *p* ≤ 0.05 was considered significant.

## Results

### *FOXM1* message is elevated in most but not all relapsed myelomas

**Fig. 1:**
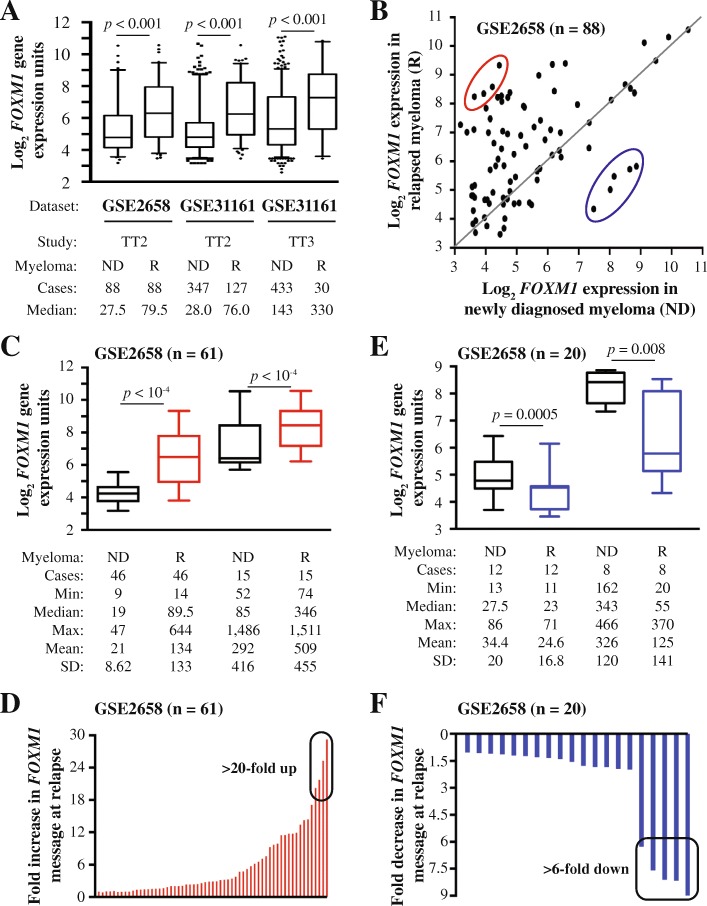
*FOXM1* message levels in paired samples of new and relapsed myeloma. **a** *FOXM1* message is elevated in relapsed myeloma in UAMS datasets. Shown are box plots of *FOXM1* mRNA levels from Total Therapy 2 (TT2) and Total Therapy 3 (TT3) datasets. For each dataset, *FOXM1* expression at baseline in newly diagnosed disease (ND) is compared to relapsed disease (R). Median *FOXM1* expression is indicated by a black horizontal line within the box. The 25th and 75th percentiles are indicated by lower and upper horizontal lines, respectively. Number of patients and median *FOXM1* expression are given at the bottom. Statistical comparisons relied on the Mann-Whitney test. **b** Dot plot of paired ND and R myelomas (*n* = 88) from the TT2 / GSE2658 dataset. Dots above (*n* = 61) and below (*n* = 20) the black horizontal line indicate up- and down-regulation of *FOXM1* at relapse, respectively. Dots on that line (*n* = 7) represent patients with no change. Four R myelomas featuring a more than 20-fold elevation of gene message and five R myelomas exhibiting a more than 6-fold drop in gene expression are denoted by red and blue ellipse, respectively. These cases are also singled out in panels D and F, respectively. **c** Source of elevated *FOXM1* at relapse. Shown is the breakdown of 61 myelomas with heightened levels of *FOXM1* at relapse in two groups distinguished by lower (left) and higher (right) baseline expression. **d** Outliers with peak expression levels at relapse. Four myelomas exhibiting increases by more than 20-fold are indicated by black ellipse. **e** Source of decreased *FOXM1* at relapse. Shown is the breakdown of 20 myelomas with reduced *FOXM1* expression at relapse in two groups distinguished by lower (left) and higher (right) baseline expression values. **f** Outliers with steep drops in gene expression at relapse. Five myelomas exhibiting decreases by more than 6-fold are indicated by black ellipse

Using the University of Arkansas for Medical Science (UAMS) Total Therapy 2 (TT2) dataset (GSE2658) as discovery tool, we recently reported that upregulation of *FOXM1* was a common feature of patients with rMM [13]. Here we confirm this finding with the help of an updated TT2 dataset that includes 127 patients with rMM and a related TT3 dataset that includes 30 patients with rMM – both available at GSE31161 (Fig. 1a). The increase in median *FOXM1* mRNA levels in these datasets, 2.7-fold in case of TT2 (76/28) and 2.3-fold in case of TT3 (330/143), was very similar to the one observed in the original TT2 dataset (2.89-fold; 79.5/27.5). Furthermore, in all 3 datasets, mean *FOXM1* expression at relapse was significantly elevated (*p* = 10^− 3^) compared to baseline. Next, we went back to the TT2 / GSE2658 dataset, presented in dot plot format in Fig. 1b, to visualize the two expression values, ND vs R, for each patient. Upregulation of *FOXM1* mRNA at relapse was seen in more than two thirds of patients (61 of 88, 69%; indicated by dots above the diagonal line), whereas *g*ene message was down in 20 (23%) patients (dots below the line) and unchanged in 7 (8%) patients (dots on the line). The magnitude of the elevation at relapse (~ 2.6-fold; 226/87.8) was similar to the decline (~ 2.3-fold; 151/64.6) using mean expression levels for comparison. These results indicated that the overall increase of *FOXM1* message at relapse was caused by the preponderance of myelomas with elevated gene expression (69%), not by the circumstance that the magnitude of elevation exceeded that of reduction.

### Outlier analysis of *FOXM1* expression in rMM

To analyze the 61 myelomas that harbored elevated *FOXM1* at relapse in greater depth, we partitioned these cases in two arbitrary groups defined by median baseline expression levels of 19 microarray units (*n* = 46) and 85 units (*n* = 15), respectively, in newly diagnosed (ND) disease (Fig. 1c, black boxes with whiskers). The increase at relapse in the low expresser group was somewhat higher (4.7-fold, 89.5/19) than in the high expresser group (4.1-fold, 346/85). The difference was due, in no small measure, to four myelomas in the low expresser group that exhibited > 20-fold elevated expression peaks at relapse. This is indicated by a black ellipse in Fig. 1d. The analysis of tumors featuring reduced *FOXM1* expression at relapse is depicted in Fig. 1e. Partitioning of the dataset (*n* = 20) in two groups with median baseline expression of 27.5 units (*n* = 12) and 343 units (*n* = 8) at the time of diagnosis (ND) demonstrated that the decrease in the high expresser group was more pronounced (6.2-fold, 343/55) than in the low expresser group (1.2-fold, 27.5/23; Fig. 1e). The difference could be attributed in large part to a subset of myelomas in the high expresser group (*n* = 5) that exhibited > 6-fold drops in gene expression at relapse (Fig. 1f, black ellipse). These findings led us to conclude that the pattern of *FOXM1* expression in rMM is heterogeneous, with outliers in both directions contributing disproportionally to the relapse-dependent shift in gene expression. To confirm the findings presented in Fig. 1 with an independent method not relying on Affymetrix arrays, we used RT-PCR to analyze sequential ND and R CD138^+^ bone marrow tumor samples from 8 patients with myeloma undergoing HDT/ASCT therapy. Although the number of clinically confirmed relapses was low (*n* = 3), the increase in *FOXM1* expression was impressive, up to 35-fold (Additional file 1: Figure S1).

### *FOXM1* promotes proliferation and drug efflux activity of myeloma cells

**Fig. 2:**
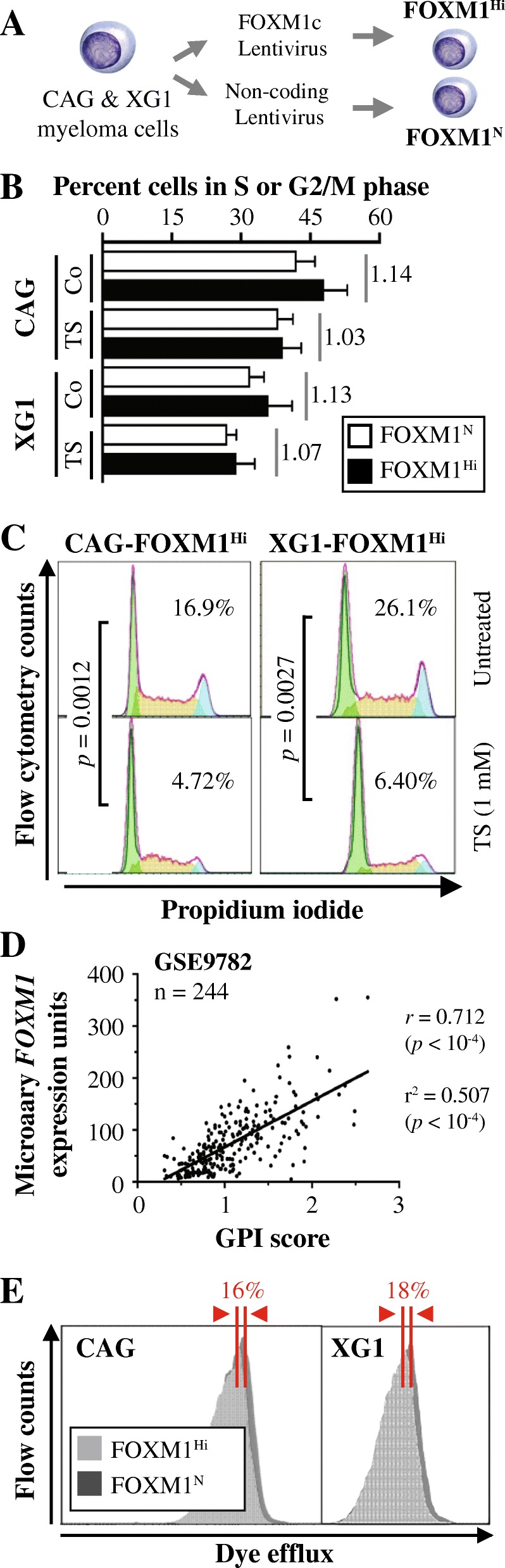
Constitutive overexpression of FOXM1 promotes growth and survival of myeloma cells in vitro. **a** Scheme of study design. FOXM1^Hi^ and FOXM1^N^ myeloma cells were generated using lentiviral gene transduction. Two HMCLs, designated CAG and XG1, were used. *FOXM1* message levels in FOXM1^Hi^ cells, relative to normal levels in FOXM1^N^ cells, were significantly elevated in both CAG cells (~ 15 fold) and XG1 cells (~ 6-fold). FOXM1 protein levels were only moderately increased: ~ 50% and ~ 30% in CAG and XG1 cells, respectively. See Figure 4a in reference [12] for details. **b** Bar diagrams depicting percent FOXM1^Hi^ and FOXM1^N^ XG1 and CAG cells in S or G2/M phase of the active cell cycle. Cells were treated with thiostrepton (TS) or left untreated for use as control (Co). The result is consistent with the analysis of cell proliferation in Figure 4b of reference [12], showing that after 1 week in cell culture, the number of viable, actively proliferating FOXM1^Hi^ cells was significantly higher than that of FOXM1^N^ cells. **c** Cell cycle fractions determined by flow cytometry. Treatment of cells using thiostrepton (TS) resulted in a significant drop in cell proliferation, using two-way ANOVA for comparison. **d** Scatter plot demonstrating positive correlation of *FOXM1* expression and myeloma proliferation in 244 Bz-treated patients from the Mayo Clinic. Tumor cell proliferation was scored with the assistance of a gene expression-based proliferation index (GPI) developed by Bergsagel et al. [20]. **e** Flow cytometric dye efflux histograms of CAG cells (left) and XG1 cells (right), demonstrating heightened ABC transporter drug pump activity in FOXM1^Hi^ cells relative to FOXM1^N^ cells. Percent differences in mean fluorescence intensity (MFI) were 16% in case of CAG and 18% in case of XG1

To assess the biological outcomes of elevated *FOXM1* in MM, we relied on two HMCLs, XG1 and CAG, as experimental model system. The cells were manipulated to contain either elevated levels of FOXM1 (FOXM1^Hi^) or normal levels of gene message and protein (FOXM1^N^) due to transfection with a human FOXM1 (isoform C) expressing lentivirus or a non-coding “empty” lentivirus (used as control), respectively [12] (Fig. 2a). Flow cytometric determination of cell proliferation showed that upregulation of FOXM1 promotes cell cycle progression (Fig. 2b). FOXM1^Hi^ CAG and XG1 cells exhibited 14 and 13% higher growth rates, respectively, compared to FOXM1^N^ cells (compare columns labeled “Co”). Treatment of cells with the FOXM1-inhibiting thiazole antibiotic, thiostrepton (TS) [17], slowed the growth of both FOXM1^Hi^ and FOXM1^N^ cells. However, the low Hi-to-N ratio indicated that FOXM1^Hi^ cells were more sensitive to TS than FOXM1^N^ cells (columns labeled “TS”). Figure 2c depicts an example of the magnitude of TS-dependent growth inhibition of FOXM1^Hi^ cells under conditions of higher drug concentration compared to panel B. The decrease in proliferation amounted to 72% (4.72/16.9) in case of CAG cells (left panel) and 76% (6.40/26.1) in case of XG1 cells (right panel). The results presented above were in line with genetic evidence gleaned from transcriptomic studies using microarrays, indicating that FOXM1 promotes myeloma proliferation. Thus, *FOXM1* expression is positively correlated (Pearson’s *r* = 0.712, *p* < 10^− 4^) with myeloma cell proliferation in 244 Bz-treated patients available at GSE9782 [18], using the global gene expression-based proliferation index (GPI) of myeloma devised by Bergsagel et al. [19] as proxy of actual tumor cell proliferation (Fig. 2d). Similarly, we recently reported [13] that *FOXM1* message levels in nMM and rMM paralleled the GPI of developed by Hose and his associates [20]. Next, we used eFluxx-ID Gold MDR analysis to demonstrate that upregulation of FOXM1 results in enhanced ABC-transporter efflux-pump activity in myeloma (Fig. 2d). This finding suggested that FOXM1-dependent promotion of cell cycle progression in myeloma may facilitate drug resistance by means of enhanced outflow of myeloma drugs.

### Enforced expression of FOXM1 lessens sensitivity to myeloma drugs in vitro

**Fig. 3:**
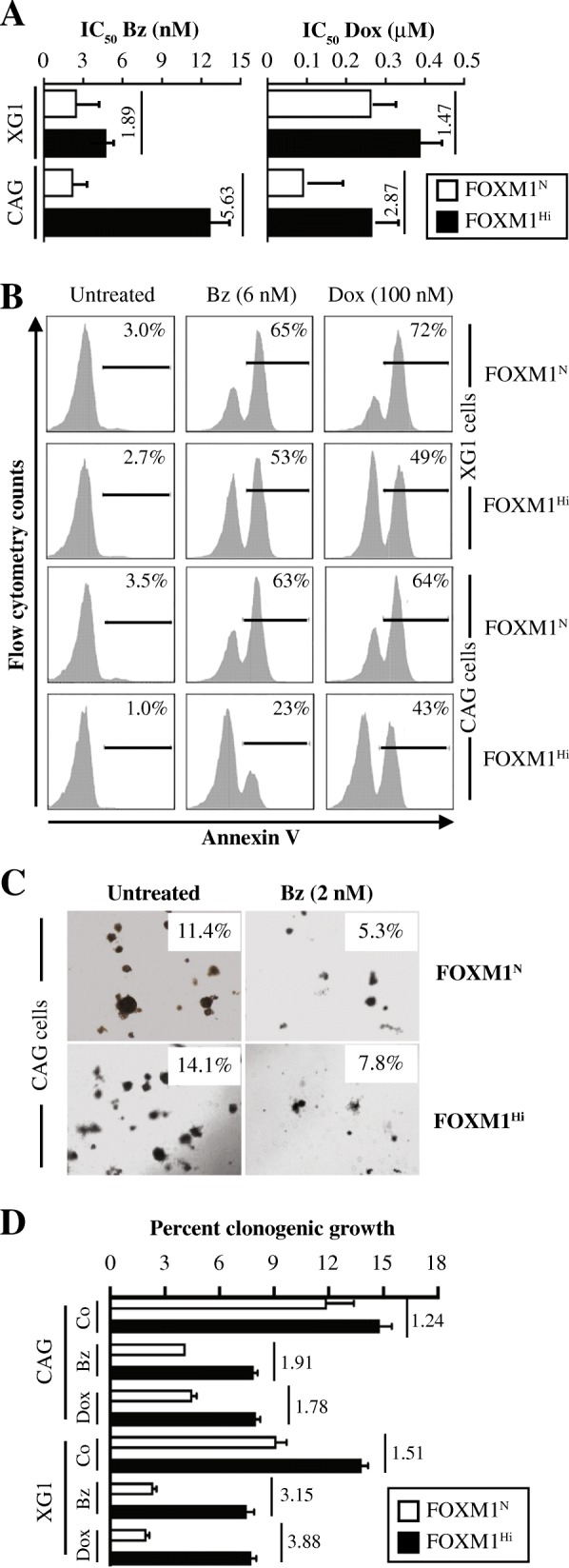
Upregulation of FOXM1 promotes drug resistance of myeloma in vitro. **a** Bar diagram depicting the half maximal inhibitory concentrations (IC_50_) of bortezomib (Bz) and doxorubicine (Dox) in XG1 and CAG myeloma cells expressing elevated (FOXM1^Hi^) or normal (FOXM1^N^) levels of the transcription factor. Cells were grown for 1 week in bulk suspension culture in presence of a log_2_ dose range of either drug. IC_50_ was determined using non-linear regression analysis of dose response curves (GraphPad Prism 7 software). Hi-to-N ratios of IC_50_ (indicated by vertical short lines and numbers) reflect the magnitude by which overexpression of FOXM1 reduced drug-induced cell killing. **b** Representative flow histograms comparing levels of drug-induced apoptosis in paired FOXM1^Hi^/FOXM1^N^ samples of XG1 (upper half) and CAG (lower half) cells treated with 6 nM Bz (center column) or 0.1 μM Dox (right column). Cells left untreated were used as control (left column). APC (allophycocyanin)-conjugated antibody to annexin V was used to determine the fraction of cells undergoing apoptosis (indicated as percentages above black horizontal lines). In all cases, death was attenuated by enforced expression of FOXM1. **c** Photographic images of soft-agar plates containing colonies of FOXM1^Hi^ and FOXM1^N^ CAG myeloma cells. Cells were treated with the indicated dose of bortezomib or left untreated. Clonogenic growth expressed as percentage of cells able to form colonies is given in the insets. **d** Bar diagram depicting soft-agar clonogenicity of FOXM1^Hi^/FOXM1^N^ CAG or XG1 cells treated with 2 nM Bz or 0.1 μM Dox. Cells left untreated were used as control (Co). The Hi-to-N ratios of the average colony numbers, determined in triplicate experiments, are indicated by vertical numbers next to vertical lines. The ratios reflect the extent by which overexpression of FOXM1 mitigated drug-dependent inhibition of clonogenic growth

Because recurrent cancers including rMM may acquire therapy resistance due to alterations in biological pathways in which FOXM1 is involved [21], we wondered whether upregulation of FOXM1 may decrease the sensitivity of myeloma cells to widely used myeloma drugs, such as bortezomib (Bz) and doxorubicin (Dox). We found that enforced expression of FOXM1 renders myeloma partially resistant to both drugs, as evidenced by half-maximal inhibitory concentrations (IC_50_) that, in case of Bz, were 5.6-fold (CAG) and 1.9-fold (XG1) higher in FOXM1^Hi^ than FOXM1^N^ cells **(**Fig. 3a**, left)**. In case of Dox, they were 2.9-fold (CAG) and 1.5-fold (XG1) higher **(**Fig. 3a**, right)**. Flow cytometric measurements of annexin V, a marker of apoptosis, were in agreement with these results as FOXM1^Hi^ cells invariably exhibited less drug-induced death than FOXM1^N^ cells did. For example, there was a 2.7-fold difference in CAG cells treated with Bz (23% vs. 63%) and a 1.5-fold difference in XG1 cells treated with Dox (49% vs. 72%, Fig. 3b). Determination of myeloma growth in soft agar demonstrated the clonogenic advantage of FOXM1^Hi^ cells. One example, depicted in Fig. 3c, shows that FOXM1^Hi^ CAG cells treated with 2 nM Bz or left untreated produced ~ 50% (right panels) or ~ 25% more colonies (left panels), respectively, than FOXM1^N^ controls. XG1 cells demonstrated greater differences based on Hi-to-N ratios (Fig. 3d), suggesting that FOXM1 protects the clonogenic growth of these cells under conditions of selective drug pressure even more effectively than that of CAG cells. These findings were of interest in light of published reports that suppression of FOXM1 may sensitize human cancer to killing by genotoxic drugs [22].

### Elevation of FOXM1 leads to reduced sensitivity of myeloma xenografts to Bz

**Fig. 4:**
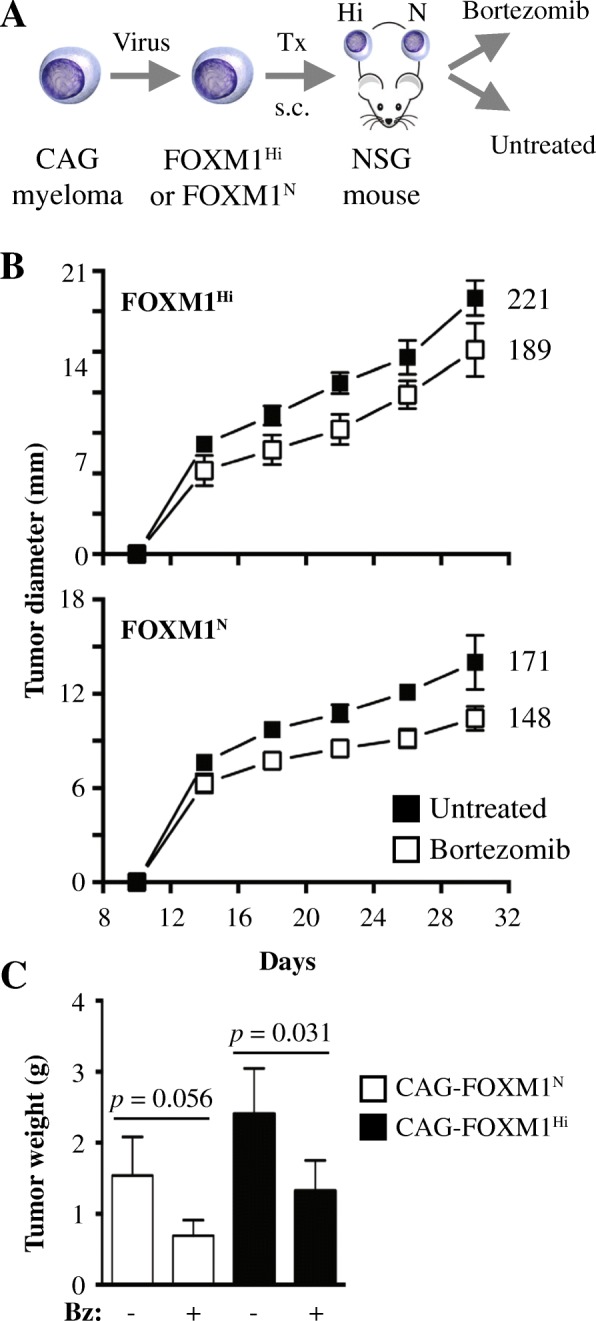
Upregulation of FOXM1 reduces susceptibility of myeloma xenografts to bortezomib in vivo. **a** Scheme of study design. FOXM1^Hi^ and FOXM1^N^ myeloma cells were generated using lentiviral gene transduction as previously described [12]. Cells were subcutaneously (s.c.) xenografted (Tx) into the right and left flank of NSG mice, respectively. Beginning on day 7 following cell transfer, 5 mice were treated with daily administrations of the proteasome inhibitor, bortezomib (Bz, 1 μg per gram body weight IV), until study termination on day 30. Five mice were left untreated. In all cases, tumor diameters were measured in 4-day intervals using a caliper, starting on day 10 after xenografting. Xenografts were harvested and weighed on a fine balance at study end. **b** Time course of tumor growth in host mice. Mean tumor diameters (squares) and standard deviations of the mean (short vertical lines with error bars) are plotted. In Bz-treated mice, the area under the curve (AUC) of FOXM1^N^ tumors (148) was 13% smaller than that of FOXM1^Hi^ (171) tumors. In untreated mice, the AUC of FOXM1^N^ tumors (189) was 14% smaller than that of the FOXM1^N^ (221) tumors. **c** Mean tumor weight at end of study. FOXM1^Hi^ xenografts treated with Bz (1.32 ± 0.434 g) were significantly smaller than their untreated counterparts (2.40 ± 0.647 g) using Mann-Whitney’s *t* test (*p* = 0.031). FOXM1^N^ xenografts showed the same trend (1.54 ± 0.543 g vs. 0.692 ± 0.217 g) but missed the 5% threshold of statistical significance (*p* = 0.056). Median tumor weights in all 4 groups were significantly different by Kruskall-Wallis analysis (*p* = 0.006)

To complement the in vitro findings described above with in vivo data, we determined whether upregulation of FOXM1 results in decreased susceptibility of myeloma to proteasome inhibition (PI). We used human-in-mouse myeloma xenografts treated with Bz or left untreated (control) as model system. FOXM1^Hi^ and FOXM1^N^ CAG cells were transferred under the skin of the left and right flank of NSG mice (*n* = 10), respectively. Treatment of 5 tumor-bearing hosts using daily IP injections of Bz commenced 7 days later. Five tumor-bearing hosts left untreated were used as controls (Fig. 4a). Tumor diameters were measured in 4-day intervals to compare xenograft growth rates. FOXM1^Hi^ tumors harvested on day 30 (study endpoint) were larger than their FOXM1^N^ counterparts in both the Bz treatment arm (15.2 ± 1.97 mm vs 10.4 ± 0.771 mm) and the control arm (19.0 ± 1.31 mm vs 14.0 ± 1.73 mm) arm (Fig. 4b). This was consistent with FOXM1’s growth-promoting activity described above. However, in terms of sensitivity to PI, FOXM1^Hi^ tumors were slightly less responsive (by 10%) than their FOXM1^N^ counterparts on the contralateral side of the same host. Specifically, Bz-dependent growth inhibition of FOXM1^Hi^ tumors at study endpoint (1.32 ± 0.434 g in treated mice vs. 2.40 ± 0.647 g in mice left untreated) amounted to 45%, whereas that of FOXM1^N^ tumors (0.692 ± 0.217 g treated vs 1.54 ± 0.543 g untreated) amounted to 55% (Fig. 4c). This result, however modest it might be, was consistent with published findings that lowering FOXM1 in human cancer cells leads to enhanced PI-induced killing [23].

### Binding of FOXM1 to Rb may promote senescence of myeloma cells

**Fig. 5:**
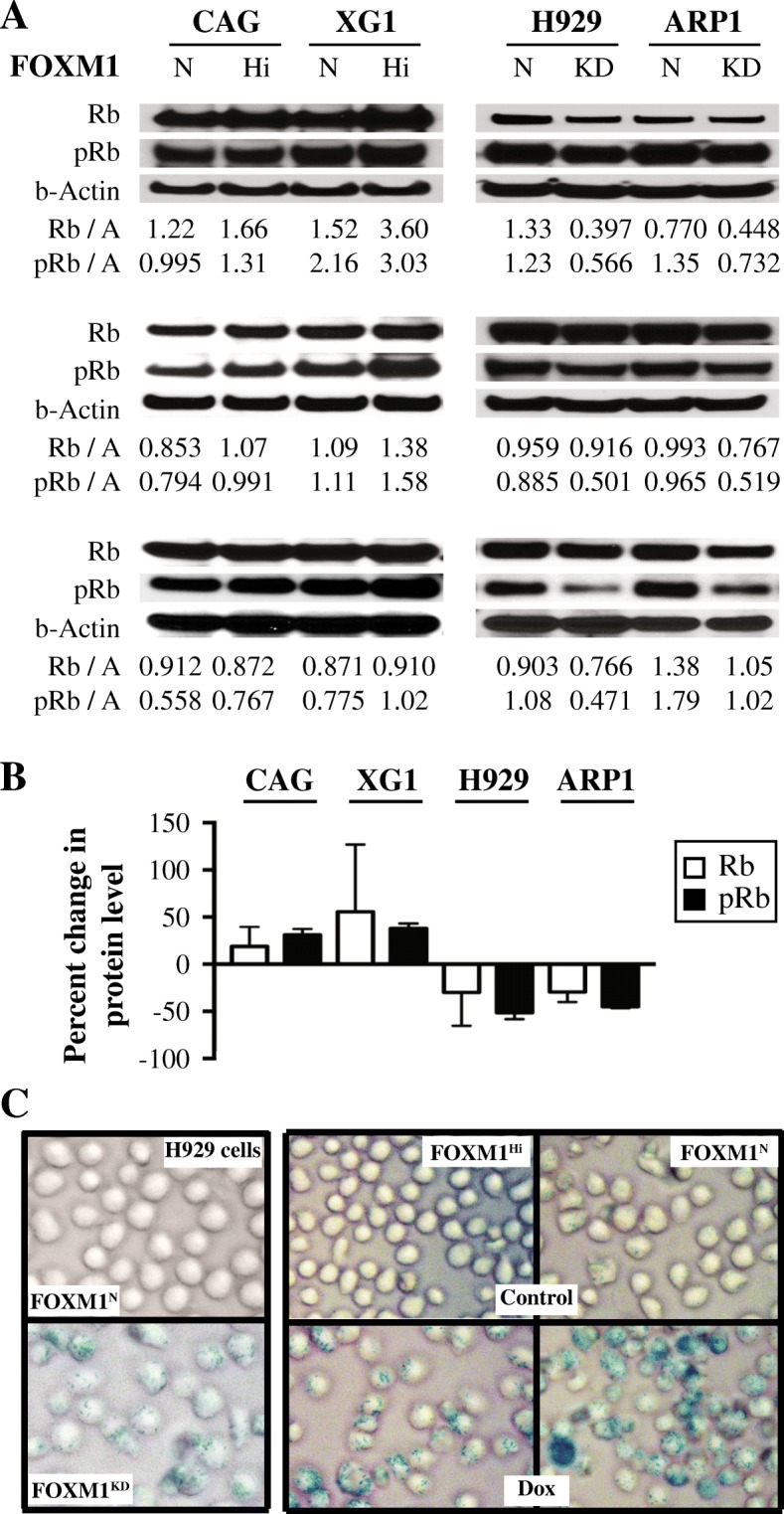
FOXM1/Rb-dependent expression of β-galactosidase in myeloma cells. **a** Co-immunoprecipitation (Co-IP) result indicating physical interaction of Rb and FOXM1 in CAG and XG1 myeloma cells. Immunoblots using a specific IgG antibody (Ab) to FOXM1 (following IP with Ab to Rb) or Rb (following IP with Ab to FOXM1) are shown on top of each other. The IgG isotype control is labeled “IgG.” Whole cell lysates not subjected to Co-IP (“Input”) were included as an additional control. **b** Shown on top is a Western blot of total Rb and its activated, phosphorylated form (pRb) in FOXM1^Hi^ and FOXM1^N^ CAG and XG1 myeloma cells. A similar blot containing samples of FOXM1^KD^ and FOXM1^N^ H929 and ARP1 myeloma cells is presented at bottom. The abundance of Rb and pRb, relative to β-actin, was determined using densitometry. The ratios are indicated below the blots. **c** The upper panel illustrates the elevation of β-galactosidase (β-gal) activity, a classic phenotype of cellular senescence, in FOXM1^KD^ H929 cells (bottom) relative to FOXM1^N^ controls (top). Cells were not treated with drug. This result was confirmed using paired FOXM1^KD^ / FOXM1^N^ ARP1 samples (not shown). Depicted in the lower panel is the increased proportion of β-gal^+^ XG1 cells following treatment with Dox. FOXM1^Hi^ cells exhibited a lesser increase than FOXM1^N^ cells (not shown). Cells were evaluated using an Olympus BX-51 Light Microscope equipped with an UPLSAPO objective (Olympus) of 40x magnification and 0.95 numerical aperture. The imaging medium was air. The light temperature of the microscope bulb varied between 3000 and 3400 K. Images were acquired with the help of a DP2 digital camera (Olympus) and DP2-BSW imaging software (Olympus), saved as TIF data files, and enhanced—with respect to brightness, contrast, and color balance—using the Adobe Photoshop CS2 Version 9.0.2 software (Adobe Systems, Inc)

Our recent work has implicated the cyclin-dependent kinase 6 (CDK6) / retinoblastoma (Rb) axis in the mechanism by which FOXM1 promotes myeloma [14]. To confirm the co-regulation of FOXM1 protein levels and the tumor suppressor, Rb, in myeloma, we performed triplicate Western analyses of paired FOXM1^Hi^ and FOXM1^N^ tumors (Fig. 5a**, left**). For sake of comparison to FOXM1^Hi^, we included FOXM1^Lo^ samples from H929 and ARP1 HMCL cells, in which FOXM1 had been knocked down using lentivirus-delivered RNA interference (Fig. 5a**, right**). Densitometric analysis of Western blots showed that total Rb and phosphorylated Rb (pRb) were increased in FOXM1^Hi^ cells by 20–40%, with somewhat higher levels seen in XG1 than CAG cells (Fig. 5b**, left**). Conversely, Rb and pRb were decreased in FOXM1^Lo^ cells (~ 30 to 50%), with the loss of the latter somewhat exceeding that of the former (Fig. 5b**, right**). Inspired by a large body of evidence that connects the Rb-governed cell fate decision of senescence with important pathways of cancer relapse such as drug resistance, tumor dormancy and tumor stemness [14], we determined whether myeloma cells might express the classic senescence-associated phenotype of β-galactosidase in a FOXM1-dependent manner. Fig. 5c**, left** shows that FOXM1 knockdown sufficed to induce β-gal activity in myeloma. Furthermore, treatment of cells using Dox caused higher β-gal^+^ scores in FOXM1^N^ than FOXM1^Hi^ cells **(**Fig. 5c**, right)**. These findings were consistent with published reports on the role of FOXM1 in suppressing cellular senescence in a variety of solid and liquid neoplasms [24].

## Discussion

The main finding of this study is experimental evidence for a role of FOXM1 in advanced myeloma. The new results implicating FOXM1 in drug-resistant disease relapse and the β-gal^+^ phenotype of cellular senescence agree with the well-known pleiotropic function of the transcription factor in cancer cell biology [25]. In both solid and liquid cancers, FOXM1 governs a wide spectrum of biological processes, including cell cycle progression, DNA damage repair, self-renewal of stem cells [26] and senescence [24] – all involved in tumor progression and the response of malignancies to cytostatic and targeted treatments [27]. In regard to diverse biological functions attributable to FOXM1 in myeloma, the transcription factor seems to resemble well-established “master” transcription factors of myeloma, such as interferon regulatory factor 4 or IRF4 (a.k.a. MUM1) [28] and the myelocytomatosis oncoprotein MYC [29]. To further put FOXM1’s role in myeloma in perspective, it is helpful to recognize that this particular member of the large forkhead box family of proteins is critically involved in the development and outcome of other B-lineage neoplasms; e.g., acute lymphoblastic leukemia [30], diffuse large cell lymphoma [31], chronic lymphocytic leukemia [32] and follicular lymphoma [33]. What is more, an overarching impact of FOXM1 on cancer as a whole has been suggested by a recent pan-cancer meta-analysis of approximately 18 thousand gene expression signatures [34], which identified the FOXM1 regulatory network as a major predictor of adverse outcomes across 39 solid and hematologic malignancies including MM.

Although the molecular mechanism by which FOXM1 promotes drug resistance in myeloma has not yet been elucidated, FOXM1-dependent increases in cell proliferation, NEK2 (NIMA related kinase 2)-dependent CIN (chromosomal instability) and ABC-transporter drug-pump activity may be involved [12]. The latter has been repeatedly implicated in drug-resistant solid cancers; e.g., in retinoblastoma [35], bladder cancer [36] and colorectal cancer [37], in which the heightened drug-efflux activity could be functionally linked to FOXM1-dependent upregulation of ABCC4, ABCG2 and ABCC10, respectively. Also playing a role may be other pathways of drug resistance that operate in solid tumors [38]; e.g., inhibition of ubiquitination-dependent FOXM1 degradation via interacting proteins, such as OTUB1 (OTU deubiquitinase, ubiquitin aldehyde binding 1) [39]; crosstalk of FOXM1 with other cellular signal transduction pathways, such as HGF / Met (hepatocyte growth factor / Met proto-oncogene, receptor tyrosine kinase) [40] and AKT (AKT serine / threonine kinase 1) [41]; and metabolic changes that effect increased oxidative defense capacity, as seen in radio-resistant head and neck squamous cell carcinoma [42]. Targeting the interactions and pathways described above – perhaps in conjunction with targeting FOXM1 directly using established [43] or emerging [44] small-drug inhibitors – may afford the re-sensitization of relapsed FOXM1^High^ myeloma to Bz and other drugs that were effective at earlier stages of myeloma therapy. A variety of molecularly targeted chemo-sensitization approaches of this sort are pursued in myeloma [45] – all attempting to build on findings in B-ALL that demonstrate that drug resistance in malignant B lymphocytes may be overcome by suppression of FOXM1 [46].

Several limitations of our study exist. First among these is the need to confirm the findings on FOXM1-dependent drug resistance in primary tumor cells. Sequential nMM → rMM samples of fractionated malignant bone marrow plasma cells obtained from patients with new and relapsed myeloma may lend themselves to that end. In this context, we should also acknowledge that our xenograft approach for testing the Bz response in vivo was not a true study of cancer relapse; instead, it merely assessed drug-dependent growth inhibition in vivo. Genetically engineered mouse models of human myeloma – in which spontaneously arising tumors can be put into remission using clinically relevant myeloma drugs, and the mice can be kept alive until relapsed tumors require salvage treatment – may mimic the situation of patients with rMM more accurately. Two validated mouse models, designated Vκ-Myc and IL6iMyc, are available for that purpose [47–50]. Another limitation of this study concerns the involvement of cellular senescence in drug-resistant myeloma. Although β-gal activity is a well-established phenotype of cellular senescence in many types of cancer including MM [51, 52], additional research is warranted to demonstrate the mechanistic link to the FOXM1-Rb pathway. Mechanisms of FOXM1-dependent senescence elucidated in neoplasms other than myeloma [53] include enhancement of Bmi-1 expression, as seen in the NIH3T3 model [54]; overexpression of miR-370, observed in AML [55]; and inhibition of the CDK4/6-FOXM1 axis by genetic means, such as enforced expression of miR-506 in ovarian cancer [56], or pharmacologic means, such as small-drug CDK inhibition in neuroblastoma [57].

## Conclusion

**Fig. 6:**
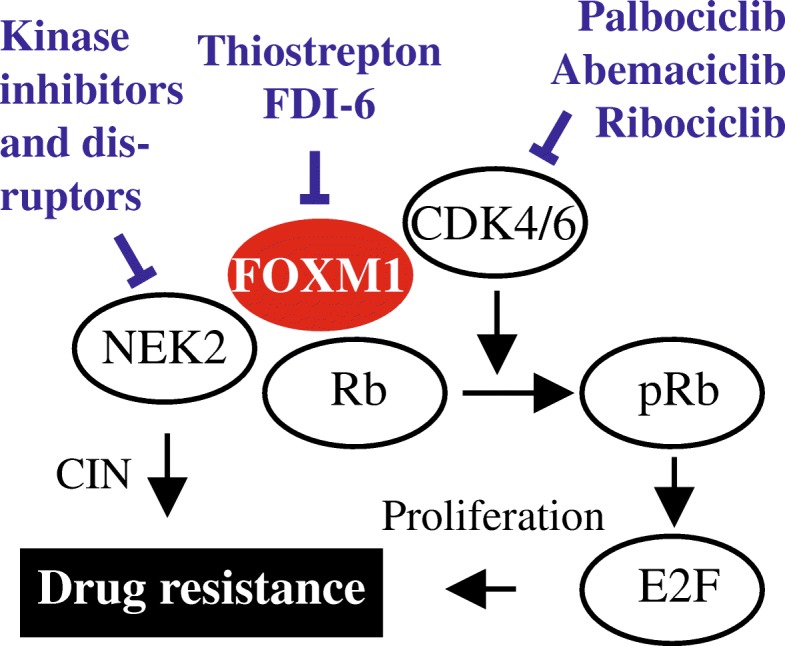
Therapeutic targeting of the FOXM1 genetic network in myeloma. FOXM1 is a proliferation-associated transcription factor that interacts in myeloma cells with the cyclin D-CDK4/6-Rb-E2F pathway, a key regulator of the G1-to-S cell cycle transition. The findings of this study demonstrate that, in addition to cell cycle progression, FOXM1 promotes drug resistance and, possibly, cellular senescence. Another interaction by which FOXM1 may desensitize myeloma to drug inhibition is NEK2. Small-molecule inhibitors of FOXM1, CDK4/6 and NEK2 are indicated. See main text for details

We now know that FOXM1 is a high-risk myeloma gene in newly diagnosed patients [12] that undergoes further upregulation in the majority of cases upon tumor relapse [13]. FOXM1’s interaction in myeloma cells with NEK2 (NIMA-related kinase 2) and the CDK4/6-Rb-E2F axis [12, 13] is of interest from a therapeutic viewpoint because CDK inhibition may be effective in myeloma [12, 58–60]. Moreover, NEK2 – a well-established transcriptional target of FOXM1 in cancer [61, 62] that has been shown to drive drug resistance in myeloma and other malignancies [63–65] – can be targeted with the help of small compounds that inhibit kinase activity [66] or trigger target degradation indirectly by means of a mechanism that involves the disruption of NEK2 binding to the kinetochore complex component NDC80 / HEC1 [67]. Figure 6 depicts the emerging FOXM1 genetic network in myeloma. Small-drug inhibitors targeting this network may overcome drug resistance in tumors in which the transcription factor is highly expressed.

## **Additional file**

Additional file 1: **Figure S1.** Quantitative, reverse transcription (RT) polymerase chain reaction (qRT-PCR) analysis of *FOXM1* gene expression in 8 myeloma patients for which 3 sequential CD138+ fractionated bone marrow tumor samples at baseline (white columns, newly diagnosed disease), initiation of HDT (high-dose therapy) and autologous hematopoietic stem cell transplantation (ASCT) therapy (grey columns) and consolidation / maintenance therapy (black columns) were available. In the course of the latter, three patiens (1, 2 and 8) and one patient (7) experienced a clinically significant and incipient FOXM1^High^ relapse, respectively. Total RNA was extracted using Quick-RNA MiniPrep (Zymo Research) and reverse transcribed using oligo dT primers and SuperScript III RT (Invitrogen). Data analysis relied on the ΔΔCt method. Primers were purchased from Integrated DNA Technologies (Coralville, Iowa). Sequences are available upon request. All increases in *FOXM1* gene expression are relative to the patient-specific baseline value, which was set at 1. All patients were consented in accordance with rules and regulations of the US Food and Drug Administration and the Declaration of Helsinki. Tumor samples were collected with institutional approval supplied with IRB 201503809 entitled “FOXM1 role in myeloma.” (PDF 1499 kb)
