# Correction to: Upregulation of FOXM1 leads to diminished drug sensitivity in myeloma

**DOI:** 10.1186/s12885-019-6443-1

**Published:** 2019-12-20

**Authors:** Chunyan Gu, Xuefang Jing, Carol Holman, Ramakrishna Sompallae, Fenghuang Zhan, Guido Tricot, Ye Yang, Siegfried Janz

**Affiliations:** 1 The Third Affiliated Hospital, Nanjing University of Chinese Medicine, Nanjing, 210023 China; 2 Key Laboratory of Acupuncture and Medicine Research, Ministry of Education, Nanjing University of Chinese Medicine, Nanjing, 210023 China; 3 Department of Pathology, The University of Iowa Roy J. and Lucille A. Carver College of Medicine, Iowa City, IA 52242 USA; 4 Iowa Institute for Genetics, The University of Iowa Roy J. and Lucille A. Carver College of Medicine, Iowa City, IA 52242 USA; 5 Department of Internal Medicine, The University of Iowa Roy J. and Lucille A. Carver College of Medicine, Iowa City, IA 52242 USA; 6 Holden Comprehensive Cancer Center, The University of Iowa Roy J. and Lucille A. Carver College of Medicine, Iowa City, IA 52242 USA; 7 Department of Medicine, Medical College of Wisconsin, Milwaukee, WI 53213 USA

**Correction to: BMC Cancer**

**https://doi.org/10.1186/s12885-018-5015-0**

As a result of an author oversight in the original article [1], the legend of Figure 5A and C is inaccurate and one panel in Figure 5C (FOXM1^N^ H929 cells shown in the top row, left) is wrong. The mistakes have now been corrected in Figure 5C appended below and in the amended legend to Figure 5A and C below. Importantly, the main text of the paper and the results and conclusions described therein are *not* affected by this correction. We sincerely apologize to the readership of BMC Cancer for any confusion the oversight may have caused.

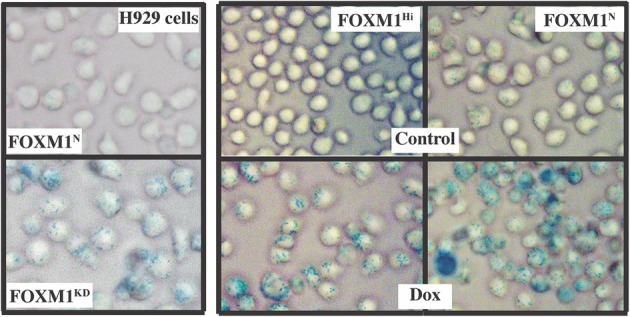



**Amended legend to Figure 5:**

**(A)** Western analysis of Rb and pRb levels in paired FOXM1^Hi^ and FOXM1^N^ myeloma cells (left) and paired FOXM1^KD^ and FOXM1^N^ myeloma cells (right).

**(C)** Depicted to the left is the elevation of β-galactosidase (β-gal) activity, a classic phenotype of cellular senescence, in FOXM1^KD^ H929 cells (bottom) relative to FOXM1^N^ controls (top). Cells were not treated with drug in both cases. The result was confirmed using paired FOXM1^KD^ / FOXM1^N^ ARP1 samples (not shown). Depicted to the right is the increased proportion of β-gal^+^ XG1 cells following treatment with Dox. FOXM1^Hi^ cells exhibited a lesser increase than FOXM1^N^ cells. Cells were evaluated using an Olympus BX-51 Light Microscope equipped with an UPLSAPO objective (Olympus) of 40x magnification and 0.95 numerical aperture. The imaging medium was air. The light temperature of the microscope bulb varied between 3000 and 3400 K. Images were acquired with the help of a DP2 digital camera (Olympus) and DP2-BSW imaging software (Olympus), saved as TIF data files, and enhanced—with respect to brightness, contrast, and color balance—using the Adobe Photoshop CS2 Version 9.0.2 software (Adobe Systems, Inc).
