# Supplementary material for: Upregulation of FOXM1 leads to diminished drug sensitivity in myeloma
Source: BMC Cancer. 2018 Nov 21;18:1152. doi: 10.1186/s12885-018-5015-0 (PMC6249818; doi:10.1186/s12885-018-5015-0)
Supplement: Supplementary file 1 — Figure S1. Quantitative, reverse transcription (RT) polymerase chain reaction (qRT-PCR) analysis of FOXM1 gene expression in 8 myeloma patients for which 3 sequential CD138+ fractionated bone marrow tumor samples at baseline (white columns, newly diagnosed disease), initiation of HDT (high-dose therapy) and autologous hematopoietic stem cell transplantation (ASCT) therapy (grey columns) and consolidation / maintenance therapy (black columns) were available. In the course of the latter, three patiens (1, 2 and 8) and one patient (7) experienced a clinically significant and incipient FOXM1High relapse, respectively. Total RNA was extracted using Quick-RNA MiniPrep (Zymo Research) and reverse transcribed using oligo dT primers and SuperScript III RT (Invitrogen). Data analysis relied on the ΔΔCt method. Primers were purchased from Integrated DNA Technologies (Coralville, Iowa). Sequences are available upon request. All increases in FOXM1 gene expression are relative to the patient-specific baseline value, which was set at 1. All patients were consented in accordance with rules and regulations of the US Food and Drug Administration and the Declaration of Helsinki. Tumor samples were collected with institutional approval supplied with IRB 201503809 entitled “FOXM1 role in myeloma.” (PDF 1499 kb) [file 12885_2018_5015_MOESM1_ESM.pdf]

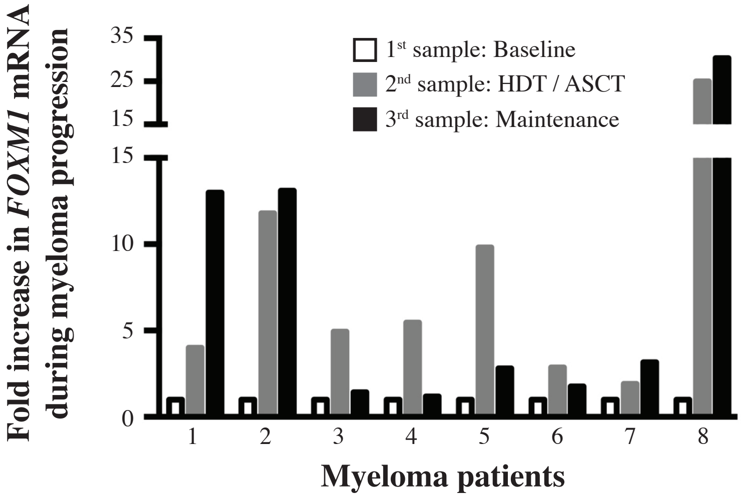

Supplemental Figure 1: Quantitative, reverse transcription (RT) polymerase chain reaction (qRT-PCR) analysis of *FOXM1* gene expression in 8 myeloma patients for which 3 sequential CD138$^+$ fractionated bone marrow tumor samples at baseline (white columns, newly diagnosed disease), initiation of HDT (high-dose therapy) and autologous hematopoietic stem cell transplantation (ASCT) therapy (grey columns) and consolidation / maintenance therapy (black columns) were available. In the course of the latter, three patiens (1, 2 and 8) and one patient (7) experienced a clinically significant and incipient FOXM1^{High} relapse, respectively. Total RNA was extracted using Quick-RNA MiniPrep (Zymo Research) and reverse transcribed using oligo dT primers and SuperScript III RT (Invitrogen). Data analysis relied on the ΔΔCt method. Primers were purchased from Integrated DNA Technologies (Coralville, Iowa). Sequences are available upon request. All increases in *FOXM1* gene expression are relative to the patient-specific baseline value, which was set at 1. All patients were consented in accordance with rules and regulations of the US Food and Drug Administration and the Declaration of Helsinki. Tumor samples were collected with institutional approval supplied with IRB 201503809 entitled "FOXM1 role in myeloma."
